# Unilateral chronic maxillary rhinosinusitis after bone maxillary graft for dental implant placement: A case report

**DOI:** 10.1002/ccr3.2760

**Published:** 2020-08-30

**Authors:** Jerome R. Lechien, Raquel Lamartine Monteiro, Stelianos Kampouridis, Rokneddine Javadian, Mihaela Horoi

**Affiliations:** ^1^ Department of Otolaryngology, Head and Neck Surgery CHU Saint‐Pierre Brussels School of Medicine Université Libre de Bruxelles Brussels Belgium; ^2^ Department of Anatomy and Experimental Oncology School of Medicine University of Mons Mons Belgium; ^3^ Department of maxillofacial Surgery CHU Saint‐Pierre Brussels School of Medicine Université Libre de Bruxelles Brussels Belgium; ^4^ Department of Radiology CHU Saint‐Pierre Brussels School of Medicine Université Libre de Bruxelles Brussels Belgium

**Keywords:** chronic, dental, graft, implant, maxillary, rhinosinusitis

## Abstract

Odontogenic chronic maxillary rhinosinusitis has to be suspected in patient with a history of dental implant placement just after bone maxillary graft.

## Graphical abstract

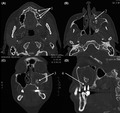

## 1 INTRODUCTION

Odontogenic chronic maxillary rhinosinusitis has to be suspected in patient with a history of dental implant placement just after bone maxillary graft. The perforation of the Schneiderian membrane through the implant placement may lead to chronic rhinosinusitis, which has to be managed by multidisciplinary team.

Chronic rhinosinusitis is one of the most common diseases around the world, accounting for 33.7 million of Americans each year, which represents 14% of the American population.^1^, ^2^ In 5%‐40% of cases of chronic maxillary rhinosinusitis (CMR), a dental etiology is found, with iatrogenic procedures (root canal treatment; 65.7%) and apical periodontal pathologies (periodontitis; 25.1%) as the main odontogenic causes.^3^ CMR due to surgical procedures performed for dental implant is a rare cause of odontogenic CMR accounting for 2%‐11% of cases.^3^, ^4^ In this paper, we reported the history of a patient with CMR due to the migration of a bone graft of the maxillary alveolar ridge, which was performed for the purpose of the placement of dental implants.

## 2 CASE

**Figure 1:**
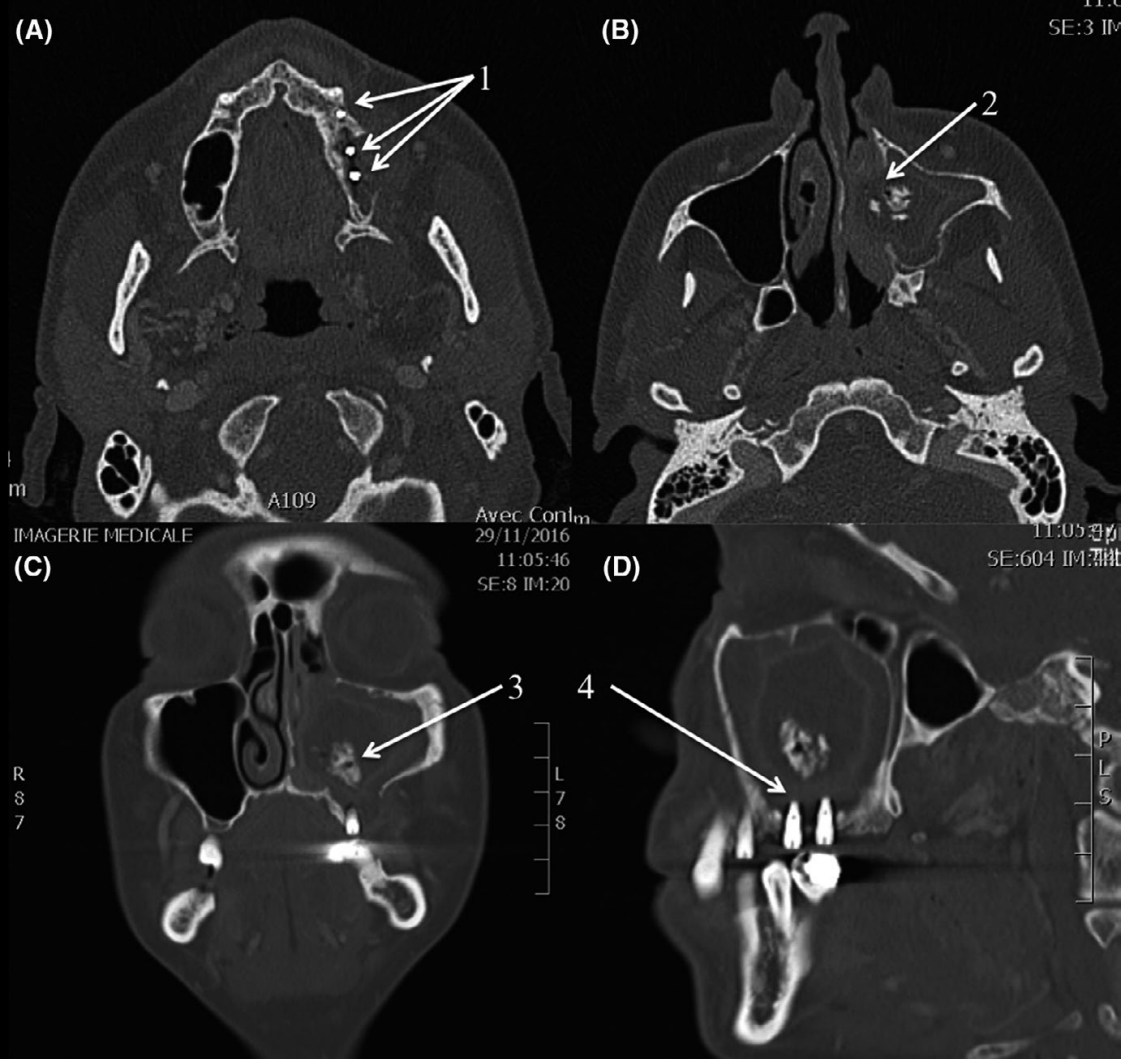
Chronic maxillary rhinosinusitis caused by bone graft migration. Preoperative axial (A, B), coronal (C), and lateral (D) CT scan section showing the three dental implants (1), the lysis of the lateral wall of the nasal fossae (2), the bone graft migration (3), and the protrusion of dental implants into the maxillary sinus (4)

A 39‐year‐old woman was referred to the Department of Otolaryngology—Head & Neck Surgery for a 2‐month history of mild left‐sided maxillary discomfort, severe left nasal obstruction, and smelly discharge. The history of the patient was characterized by tooth loss in the second quadrant (25 and 26). Patient benefited from the placement of dental implants 3 months before the consultation. First, the surgeon proceeded to the placement of a bone graft for a sinus lift, and, a couple of days later, the placement of three dental implants in the new grafted bone. At this time, the implants were correctly placed regarding the surgeon. According to the anamnesis, the patient benefited from the surgery in an emerging country (medical tourism). The postoperative imaging showed the correct position of the dental implants. One month after the surgical procedures, the patient developed the first rhinosinusal symptoms. The maxillofacial examination showed a giant red mass of the maxillary alveolar ridge in the area of the previous graft (second quadrant, teeth 25 and 26). Flexible rhinofibroscopy revealed a total nasal obstruction of the left side with a bulging of the lateral wall of the nasal fossae. The computed tomography (CT) reported a left maxillary rhinosinusitis, the lysis of the left lateral bone wall, and the migration of the alveolar bone graft into the maxillary sinus (oroantral fistula due to bone defect; Figure 1). The giant red mass in the oral cavity consisted of the bulging of the Schneiderian membrane. The surgical treatment of the patient consisted of: (a) the removal of bone graft from the maxillary sinus through middle meatotomy (functional endoscopic sinus surgery approach; FESS), (b) the washing of the maxillary sinus cavity, (c) the removal of two of the three dental implants, (d) the closure of the oroantral fistula with a Bichat flap. These procedures were made in the same operative time. Regarding the bacterial analysis of the content of the maxillary sinus (polymicrobial: *Streptococcus, Fusobacterium*), patient received postoperative antibiotic therapy for 10 days (empirical amoxi/clav that was maintained). The rest of the postoperative treatment includes nasal saline solution (6/d) and corticosteroid spray (2/d) for 6 weeks. The third implant was well osteointegrated and, thus, not removed. The patient did not want to have another reconstruction, and she opted for removable prosthesis.

At the end of the management of the patient and regarding the rare situation of this case, we decided to publish the history of the patient. Thus, we have invited the patient for a free consultation to complete the medical history and the informed consent for the publication. During the consultation, the physician was surprised by history of the patient, which was characterized by many unusual medical events. First, during adolescence, she explained that she easily damaged the teeth when she ate hard foods (ie, baguette, well‐cooked meat, etc) and she had many orthopedic traumas. Second, the anamnesis revealed the occurrence of many urinary lithiasis requiring a nephrectomy. Third, the clinical examination reported an important spine and chest deformation. With regard to the unusual clinical picture, we addressed patient to the department of internal medicine for the suspicion of a bone disease (calcium and phosphorus metabolism). A bone scan was realized and reported osteopenia, while chest imaging exhibited important scoliosis with deformation of chest cavity (asymmetric deflection of sternum and the left side of chest). However, the biology was normal. The diagnosis of an idiopathic form of early osteopenia was retained, and patient was carefully followed in the department of bone disease. One year after the surgery, the follow‐up of patient was unremarkable.

## 3 DISCUSSION

Dental implant is a routine surgical approach for the replacement of missing teeth. The most important success factors for the surgery are the bone density, the thinness of edentulous alveolar ridges, and bone quality.^5^ Moreover, surgeon should be careful about the presence of favoring factors of bone weakness such as osteopenia/osteoporosis, and calcium and phosphorus metabolism diseases. Inadequate maxillary bone thickness may be associated with protrusion or migration of implant(s) into the maxillary sinus,^6^ which is reported in 2%‐11% of cases of CMR.^3^, ^4^ Thus, patients with a too thin/lacking maxillary bone floor may benefit from the realization of sinus lift bone graft allowing the osteointegration of dental implant(s) at least 3‐6 months after the graft surgery.^7^ In the present case, the placement of the implants only 2 days after the bone graft procedure had undoubtedly impaired the graft consolidation that has been propelled into the maxillary sinus. To date, only a few cases of CMR caused by maxillary sinus floor lift,^8^ surgical graft procedure,^8^ or migration of dental implant(s) into the maxillary sinus 3, 9, 10, 11 have been reported.

Clinically, the timeline of the development of both signs and symptoms could reflect the progressive migration of the graft into the sinus. The migration is associated with a lesion of the Schneiderian membrane, affecting the sinus homeostasis and leading to mucosal inflammation and obstruction of the middle meatus. The diagnosis of odontogenic CMR is based on the clinical history, findings, and the realization of a CT scan.^4^ The CT scan may characterize the oroantral fistula, the position of the dental implants, the migration of the foreign body into the sinus, the chronic mucous swelling associated with a reaction to foreign body, and the potential bone lysis related to the chronic inflammatory process as found in our patient.^12^ Traditionally, hyperdense component into the maxillary sinus may be related to many etiologies including ectopic tooth fragment, endodontic material, calcified cyst or tumor, osteoma, condensing osteitis, fungal ball(s), cancer, or metastasis.^4^, ^13^ In the present case, the diagnosis was obvious with respect to the clinical history.

The odontogenic CMR treatment is based on both medical and surgical approaches. Starting with the close of the oroantral fistula allows the control of the infection origin. Addressing the sinusal component with FESS allows the removal of the foreign bodies with a curved aspiration or a curved forceps and the opening of the sinus cavity for an adequate drainage. FESS is safe, quick, and remains a minimal invasive surgery associated with less bleeding and a shorter hospitalization time.^4^ In a second time, maxillofacial surgeon may proceed to the replacement of the sinus bone graft and dental implant(s). Medical treatment is based on decongestants and empirical antibiotics, which were selected with bacterial cultures in a second step. In the present case, the implant fixture was removed because the exposed length into the maxillary sinus exceeded 5 mm. Indeed, some reports indicated that implant exposure greater than 4 mm from the maxillary sinus floor may lead to rhinosinusitis.^14^ Another problem raised by our case concerns the responsibilities of the dental surgeon who placed both the bone graft and the dental implants. An adequate workup before the placement of dental implants may help the physician to avoid complication. A proper follow‐up of the patient is required after the implant placement to quickly detect some complications.^15^

Another interesting point in the presentation of this case concerns the context of the discovery of the early form of osteopenia. In practice, it remains impossible to perform complete internal medicine anamnesis for each patient. The unusual cause of chronic rhinosinusitis and the wish to publish the patient history led to the discovery of an unexpected disease. It is highly probable that if the physician did not want to publish the history of patient, the bone disease was not discovered.

## 4 CONCLUSION

Although CMRs due to migration of bone maxillary graft are very rare, it must be suspected in patients with history of placement of dental implant(s) after sinus lift. The history of patient is still crucial for suspecting the CMR etiology. Dental surgeons performing similar procedures should be aware of the possible complications that can arise from foreign debris invading the maxillary sinus. Moreover, in rare cases, the interest to publish a case report may lead to the discovery of some interesting findings in the patient that can improve the management of patient.

## CONFLICT OF INTEREST

None declared.

## AUTHOR CONTRIBUTIONS

JR: wrote the paper, analyzed the case, conducted literature review, and performed the research. MH and RL: analyzed the case and wrote the part “case report.” SK (radiologist): provided the images relating to the case and corrected a large part of the paper (clinical arguments and English language). MH and RJ: coordinated and corrected the paper.
